# New Strategies to Promote Macrophage Cholesterol Efflux

**DOI:** 10.3389/fcvm.2021.795868

**Published:** 2021-12-23

**Authors:** Hong Y. Choi, Isabelle Ruel, Shiwon Choi, Jacques Genest

**Affiliations:** Cardiovascular Research Laboratories, Research Institute of the McGill University Health Center, Montreal, QC, Canada

**Keywords:** macrophages, cholesterol, ATP binding cassette A1 transporter, desmocollin 1, docetaxel

## Abstract

The capacity of macrophages to dispose of cholesterol deposited in the atherosclerotic plaque depends on their ability to activate cholesterol efflux pathways. To develop athero-protective therapies aimed at promoting macrophage cholesterol efflux, cholesterol metabolism in THP-1 monocyte-derived macrophages has been extensively studied, but the intrinsic sensitivity of monocytes and the lack of a standardized procedure to differentiate THP-1 monocytes into macrophages have made it difficult to utilize THP-1 macrophages in the same or similar degree of differentiation across studies. The variability has resulted in lack of understanding of how the differentiation affects cholesterol metabolism, and here we review and investigate the effects of THP-1 differentiation on cholesterol efflux. The degree of THP-1 differentiation was inversely associated with ATP binding cassette A1 (ABCA1) transporter-mediated cholesterol efflux. The differentiation-associated decrease in ABCA1-mediated cholesterol efflux occurred despite an increase in ABCA1 expression. In contrast, DSC1 expression decreased during the differentiation. DSC1 is a negative regulator of the ABCA1-mediated efflux pathway and a DSC1-targeting agent, docetaxel showed high potency and efficacy in promoting ABCA1-mediated cholesterol efflux in THP-1 macrophages. These data suggest that pharmacological targeting of DSC1 may be more effective than increasing ABCA1 expression in promoting macrophage cholesterol efflux. In summary, the comparison of THP-1 macrophage subtypes in varying degrees of differentiation provided new insights into cholesterol metabolism in macrophages and allowed us to identify a viable target DSC1 for the promotion of cholesterol efflux in differentiated macrophages. Docetaxel and other pharmacological strategies targeting DSC1 may hold significant potential for reducing atherogenic cholesterol deposition.

## Introduction

Atherosclerosis is a major cause of cardiovascular disease in humans and atherosclerotic cardiovascular diseases (ASCVD) including heart attack, stroke and peripheral artery disease represent the leading cause of morbidity and mortality worldwide (1). The pathogenesis of atherosclerosis is a complicated process involving multiple factors, signaling pathways and cell types: infiltration of cholesterol-rich and apoB-containing lipoproteins such as low-density lipoproteins (LDLs) into the subendothelial space of arteries, LDL retention within the arteries by extracellular matrix proteoglycans secreted by arterial smooth muscle cells, chemical and enzymatic modifications of LDLs that render LDLs pro-inflammatory and immunogenic, activation of endothelial cells and recruitment of immune cells to the arteries, differentiation of recruited monocytes into phagocytic macrophages, formation of macrophage foam cells by taking up modified LDLs *via* scavenger receptors, foam cell death and generation of lipid-rich necrotic cores, recruitment of more immune cells, and development into a chronic inflammatory disease (2, 3). The formation of macrophage foam cells in the artery wall is considered to be the hallmark of early atherosclerosis lesions.

Macrophages play as the first line of defense against the development of atherosclerosis by reducing the subendothelial buildup of modified extracellular LDLs and facilitating cholesterol outflow through their cholesterol processing capacity that involves uptake, esterification, storage and efflux of cholesterol. However, persistent hypercholesterolemic conditions impair the cholesterol processing capacity, leading to the generation of cholesterol-overloaded dysfunctional or malfunctional macrophages that drives atherogenesis by activating inflammatory reactions and apoptotic pathways (4, 5). Cholesterol metabolism in macrophages is tightly associated with cellular events such as pro- or anti-inflammatory polarization, phagocytosis and autophagy (6–8). Cholesterol homeostasis is therefore vital for macrophage function and viability.

Cellular cholesterol homeostasis is exquisitely regulated at several levels, including influx, biosynthesis, storage and efflux of cholesterol. Persistent hypercholesterolemia and unregulated uptake of modified LDLs *via* scavenger receptors make it challenging for macrophages in the atherosclerotic plaque to maintain cholesterol balance. To deal with the influx of cholesterol, macrophages have developed multiple cholesterol efflux pathways (9, 10). In the absence of a cholesterol catabolic pathway in macrophages, the efflux pathways play key roles in maintaining cholesterol homeostasis and are attractive targets for the development of drugs to treat ASCVD.

## Cholesterol Efflux Pathways in Macrophages and Therapeutic Targets

**Figure 1:**
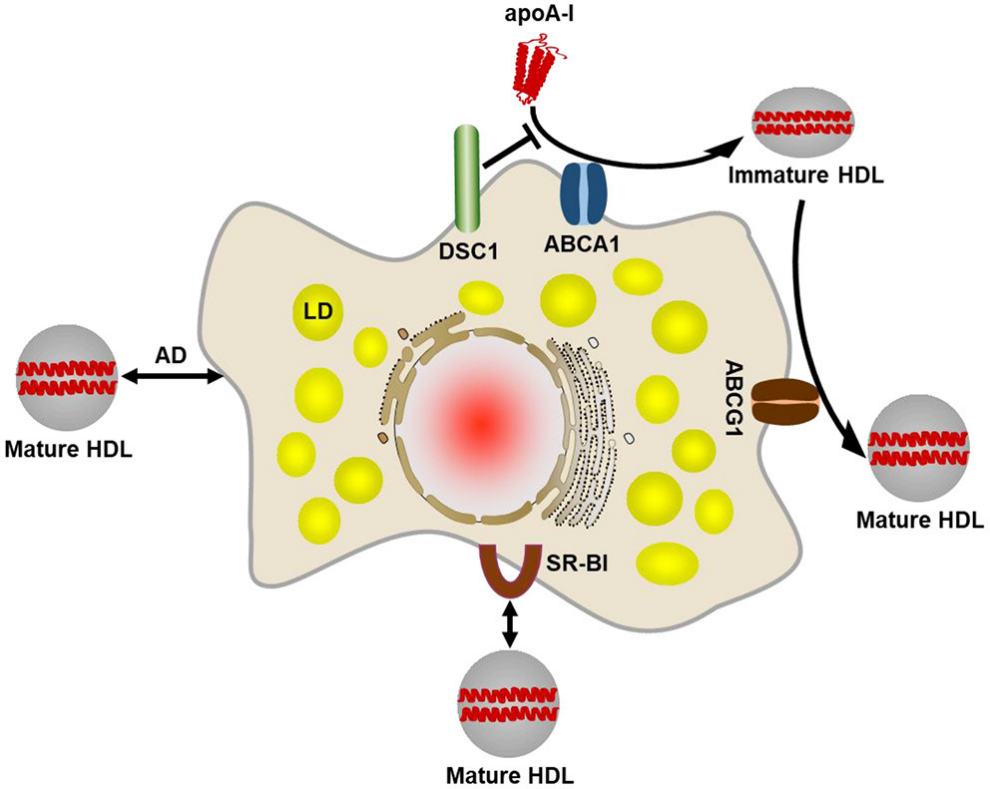
Four main cholesterol efflux pathways in macrophages. The ABCA1-mediated cholesterol efflux pathway plays the primary role in removing excess cholesterol from macrophages. ABCA1 creates a plasma membrane microdomain to facilitate efflux of phospholipids and cholesterol to lipid-free or lipid-poor apolipoproteins such as apoA-I. An apoA-I binding protein DSC1 sequesters apoA-I and prevents the lipidation of apoA-I by ABCA1. The lipidated apoA-I is called a nascent HDL particle. The nascent, immature HDL acquires additional cholesterol via the ABCG1-mediated efflux pathway so as to form a mature HDL particle. It seems likely that ABCG1 function is to increase the pool of cholesterol available for efflux. Mature HDL particles bind to SR-BI on the cell surface, which facilitates diffusion of cholesterol molecules between the plasma membrane and the HDL particle bound. The extracellular domain of SR-BI forms a non-polar channel to mediate the cholesterol exchange. Mature HDL particles are also capable of acquiring cholesterol via aqueous diffusion (AD). AD is a simple diffusion process in which cholesterol molecules desorb from the plasma membrane or HDL particles, move down its concentration gradient in the extracellular aqueous space, and incorporate into the plasma membrane or HDL particles. LD, lipid droplet.

While there is evidence that circulating macrophage foam cells can be sequestered in the spleen and die by apoptosis, most arterial resident macrophages are tightly bound to the atherosclerotic plaque. Excess cholesterol accumulated in arterial macrophages is transported to the liver for disposal or recycling of cholesterol, known as reverse cholesterol transport (RCT) (11). The initial and most crucial step in macrophage RCT is efflux of cholesterol from the cell to extracellular acceptors (12). There are four main cholesterol efflux pathways in macrophages: (a) ATP binding cassette A1 (ABCA1) transporter-mediated efflux, (b) ATP binding cassette G1 (ABCG1) transporter-mediated efflux, (c) Scavenger receptor class B type I (SR-BI)-mediated efflux, and (d) aqueous diffusion (Figure 1) (9, 10, 13).

Pathogenic genetic variants at the *ABCA1* transporter gene are the cause of Tangier disease, a rare form of severe high-density lipoprotein (HDL) deficiency and ABCA1 is the rate-limiting factor in the formation of HDL particles (14–17). HDL particles are formed in a process of removing excess cellular cholesterol and the process is termed as HDL biogenesis. The ABCA1 is upregulated in cholesterol-loaded cells and creates a plasma membrane (PM) microdomain for cholesterol removal. Extracellular lipid acceptors such as apolipoprotein (apo) A-I interact and solubilize phospholipids and cholesterol in the microdomain, producing apoA-I-lipid complexes called nascent HDL particles (18, 19). ABCA1-mediated cholesterol efflux is not specific for only apoA-I but can also occur with apoA-II, apoA-IV, and apoE (20, 21). In cholesterol-loaded macrophages, ABCA1-mediated efflux is the major pathway to remove cholesterol (22, 23).

The nascent, immature HDL particles are matured by taking up more cholesterol *via* ABCG1 (24). Although no genetic disease caused by ABCG1 mutations has been reported, decrease in ABCG1 expression levels was observed in metabolic disorders (25, 26). The working mechanism of ABCG1-mediated cholesterol efflux has not been elucidated. One model proposes that ABCG1 may promote protrusion of cholesterol from the PM to facilitate uptake by HDL particles (27). Another model proposes that ABCG1 may rearrange phospholipids in the PM such that the membrane diffuses cholesterol into the outer leaflet where HDL particles can take it up (27).

The SR-BI is an HDL receptor that binds mature HDL with high affinity. Unlike ABCA1- and ABCG1-mediated unidirectional cholesterol flux, SR-BI mediates cholesterol influx as well as efflux (13, 28, 29). The direction of cholesterol flux is determined by the concentration gradient of cholesterol between the PM and the HDL particle bound. When cholesterol concentrations in HDL particles are higher than in the PM, SR-BI drives cholesterol influx into the cell. Because of the unique function of SR-BI in mediating the bidirectional flux of cholesterol, it is difficult to clarify whether SR-BI contributes to the net cholesterol efflux in macrophages. It may depend on various factors such as cholesterol concentrations, the expression levels of SR-BI and other lipid transporters, the availability of HDL particles, and the metabolic status of a given cell.

Aqueous diffusion, also called as passive diffusion, is another pathway that allows a bidirectional exchange of cholesterol between HDL particles and the PM. Cholesterol has a limited but finite solubility in water in the range of 10 nM, which allows cholesterol molecules to move through the aqueous phase and down their concentration gradient between HDL particles and the PM. Although no lipid transporter is involved in the pathway, there are various influencing factors such as the rate of cholesterol desorption from the PM, the distribution of cholesterol among PM microdomains, and HDL characteristics including size, structure and lipid composition (9, 13). ABCG1-mediated enrichment of cholesterol in the outer leaflet of PM may also contribute to aqueous diffusion (30).

As the movement of cholesterol in the bidirectional exchange pathways depends on the concentration gradient of free cholesterol, free cholesterol-rich HDL particles can become dysfunctional or malfunctional by playing as a cholesterol donor in the atherosclerotic plaque (31). The atherogenic movement of free cholesterol from HDL to macrophages may be avoided by accelerating the egress of HDL particles from the plaque, but our knowledge on the HDL egress route is still very limited. With our current understanding, it is difficult to identify molecular targets to promote net cholesterol efflux through the bidirectional cholesterol trafficking pathways. Thus, the ABCA1- and ABCG1-mediated cholesterol efflux pathways have been extensively studied for the development of drugs promoting cholesterol efflux. The key players in the pathways, ABCA1, ABCAG1, apoA-I, and apoE have been intensively investigated as therapeutic targets, but it has proven elusive to find agents that are clinically useful and specifically enhance their cholesterol efflux activity. The agents that have been evaluated include apoA-I and apoE mimetics, recombinant HDL and HDL-like particles, HDL metabolism regulators such as niacin and inhibitors of cholesteryl ester transfer protein, and the modulators of ABCA1, ABCAG1, apoA-I, and apoE expression at the transcription, post-transcription, translation and post-translation levels (32, 33).

The failure of current approaches is due in part to our incomplete understanding of cholesterol metabolism and interconnected complex metabolic networks. In the course of identifying molecular components involved in the ABCA1-mediated cholesterol efflux pathway, we have discovered that desmocollin 1 (DSC1) localized in a cholesterol-rich PM microdomain inhibits the ABCA1-mediated pathway (Figure 1) (34). A member of the desmosomal cadherin family, DSC1 conserves cholesterol in the PM microdomain by inhibiting ABCA1-mediated cholesterol efflux. In the absence of clinically useful therapy to increase macrophage cholesterol efflux, the identification of DSC1 as a negative regulator of the ABCA1 pathway opens new doors to the development of therapeutic agents. Inhibitors are superior to stimulators in achieving specific and selective pharmacological targeting. Therefore, pharmacological inhibition of DSC1 may be more effective than strategies used to stimulate the expression of ABCA1, ABCG1, apoA-I and apoE. Another advantage of targeting DSC1 is that the extracellular domain of DSC1 is responsible for the inhibition of ABCA1-mediated cholesterol efflux (34, 35). Macrophages have evolved to recognize, ingest and break down foreign substances in order to initiate immune responses to them. As the phagocytic activity makes it challenging to deliver intact and active drugs into macrophages, the development of therapeutic agents targeting extracellular molecules is easier than those targeting intracellular molecules. In addition, ABCA1-mediated cholesterol efflux is the major pathway to remove excess cholesterol from macrophages, DSC1 may therefore constitute an important therapeutic target to reduce ASCVD by promoting macrophage cholesterol efflux.

## THP-1 Cells as a Model of Human Monocyte-Derived Macrophages

The major source of tissue-resident macrophages is circulating monocytes derived from hematopoietic stem cells. Monocytes recruited into various tissues differentiate into macrophages in response to tissue-specific environmental signals (36). The THP-1 monocytic cell line has been most widely used to study human monocyte and macrophage biology. THP-1 monocytes can be differentiated into macrophage-like cells that resemble human peripheral blood mononuclear cell-derived macrophages in phenotype and function (37). Among monocyte differentiation-inducing agents, phorbol 12-myristate 13-acetate (PMA) has been most effective in stimulating THP-1 monocytes to acquire macrophage features such as adherence, phagocytosis, and expression of macrophage markers (37–39). The PMA-differentiated THP-1 cells have been commonly used to study human macrophage activities, but the protocols to induce THP-1 monocytes-to-macrophage differentiation are highly variable with PMA concentrations used (5–400 ng/ml), the duration of PMA treatment (1–5 days), and the resting period after PMA treatment (0–5 days) (39–55). The degree of differentiation may therefore vary significantly across the study protocols. Considering that the differentiation is a process of changing cellular functions and that a central role of macrophages in the atherosclerotic plaque is to deal with excess cholesterol, it will be necessary to examine if there is any relationship between the degree of differentiation and cholesterol efflux capacity.

The differentiation of monocytes into macrophages is accompanied by major changes in lipid metabolism. The expression of LDL receptor is down-regulated, whereas the expression of scavenger receptors is up-regulated (56, 57). A variety of scavenger receptors including scavenger receptor class A type I and II, CD 36, lectin-like oxidized LDL receptor-1 are expressed in macrophages (58–61). In contrast to the LDL receptor that is under tight metabolic control (62), scavenger receptors are not feedback-regulated by cellular cholesterol content. Scavenger receptor-mediated, unregulated uptake of modified LDL particles such as oxidized LDL, acetylated LDL, glycated LDL, and enzymatically altered LDL drives accumulation of cholesterol in macrophages and foam cell formation (63–67). The expression of very-low-density lipoprotein (VLDL) receptor is also upregulated during monocyte-macrophage differentiation and VLDL receptor-mediated uptake of VLDL and intermediate-density lipoprotein particles contributes to the formation of macrophage foam cells (68, 69). To store cholesterol in cytoplasmic lipid droplets, the expression of acyl coenzyme A: cholesterol acyltransferase-1 is increased during the differentiation (70, 71). To maintain the storage capacity by removing excess cholesterol, cholesterol efflux factors such as apoE, ABCA1, and ABCG1 are also upregulated (13, 23). The balance between cholesterol uptake, storage and efflux is crucial for maintaining cholesterol homeostasis in macrophages. Under chronic hypercholesterolemic conditions, the balance is disturbed as excessive and unregulated uptake *via* scavenger receptors outweighs cellular capacity of storage and efflux. When the homeostatic mechanisms are imbalanced by over-uptake, macrophages activate inflammatory signaling pathways that regulate the production of inflammatory mediators such as reactive oxygen species, cytokines and chemokines. The mediators induce LDL oxidation, endothelial cell activation and monocyte recruitment (72–74). Newly recruited monocyte-derived macrophages participate in cholesterol removal processes, and thus the inflammatory response functions to sense and adapt to cholesterol-rich environments. As extracellular cholesterol is a source of cholesterol crystal formation, cholesterol uptake and storage by macrophages are athero-protective actions. However, sustained cholesterol burden creates a vicious cycle of cholesterol accumulation—inflammation—recruitment of monocyte-derived macrophages, which ultimately leads to macrophage death, necrotic core formation, and atherosclerotic lesion development. These data suggest that the altered expression of lipid metabolic genes in differentiated macrophages sets the stage for the vicious cycle.

All the above-mentioned changes in the expression of genes involved in macrophage cholesterol metabolism and inflammation have been observed in PMA-differentiated THP-1 cells (56, 57, 68, 71, 75–78) and cholesterol-loaded THP-1 cells are transformed into foam cells (50, 68, 78, 79), which has validated PMA-differentiated THP-1 cells as an excellent model to study macrophage cholesterol metabolism. It is not feasible to obtain primary tissue macrophages from the atherosclerotic plaque. There are several limitations with using primary human monocyte-derived macrophages, such as significantly variable monocytes among blood donors, difficulty of obtaining a large quantity of monocytes repeatedly from same donors, and ethical constraints. The use of PMA-differentiated THP-1 cells have allowed us to circumvent these restrictions, and the cells have become the most widely used model for primary human macrophages. Nonetheless, the PMA-induced differentiation does not entirely reproduce the differentiation state of primary monocyte-derived macrophages, thus the biological relevance of experimental results obtained from PMA-differentiated THP-1 cells should be carefully interpreted. A major issue with the PMA-induced differentiation is the lack of a standardized protocol, which makes it difficult to compare one study with another as the degree of THP-1 differentiation in one study may differ from the other. As the differentiation degree is a key determinant of macrophage phenotype and function, it is urgent to develop a gold standard protocol for THP-1 differentiation using PMA.

## Targeting Dsc1 to Promote Macrophage Cholesterol Efflux

Monocytes are highly plastic and heterogeneous, which is essential for monocytes to monitor environmental changes and to adapt their functional phenotype to a given environment. The innate environmental sensitivity makes it difficult to use the same or similar monocytes across various experimental settings and different laboratories, which is one of the reasons why it is challenging to establish a gold standard protocol for the THP-1 differentiation. Indeed, the effects of culture conditions on the responsiveness of THP-1 monocyte to PMA have been reported: THP-1 monocytes cultured at a high density for 1 month were heterogeneous in morphology and responded to PMA induction, whereas THP-1 monocytes cultured at a low density for 1 week were homogenous in morphology and did not respond to PMA (46). The results suggest that the high-density culture environment sensitizes THP-1 monocytes to differentiate into macrophages upon PMA stimulation.

**Figure 2:**
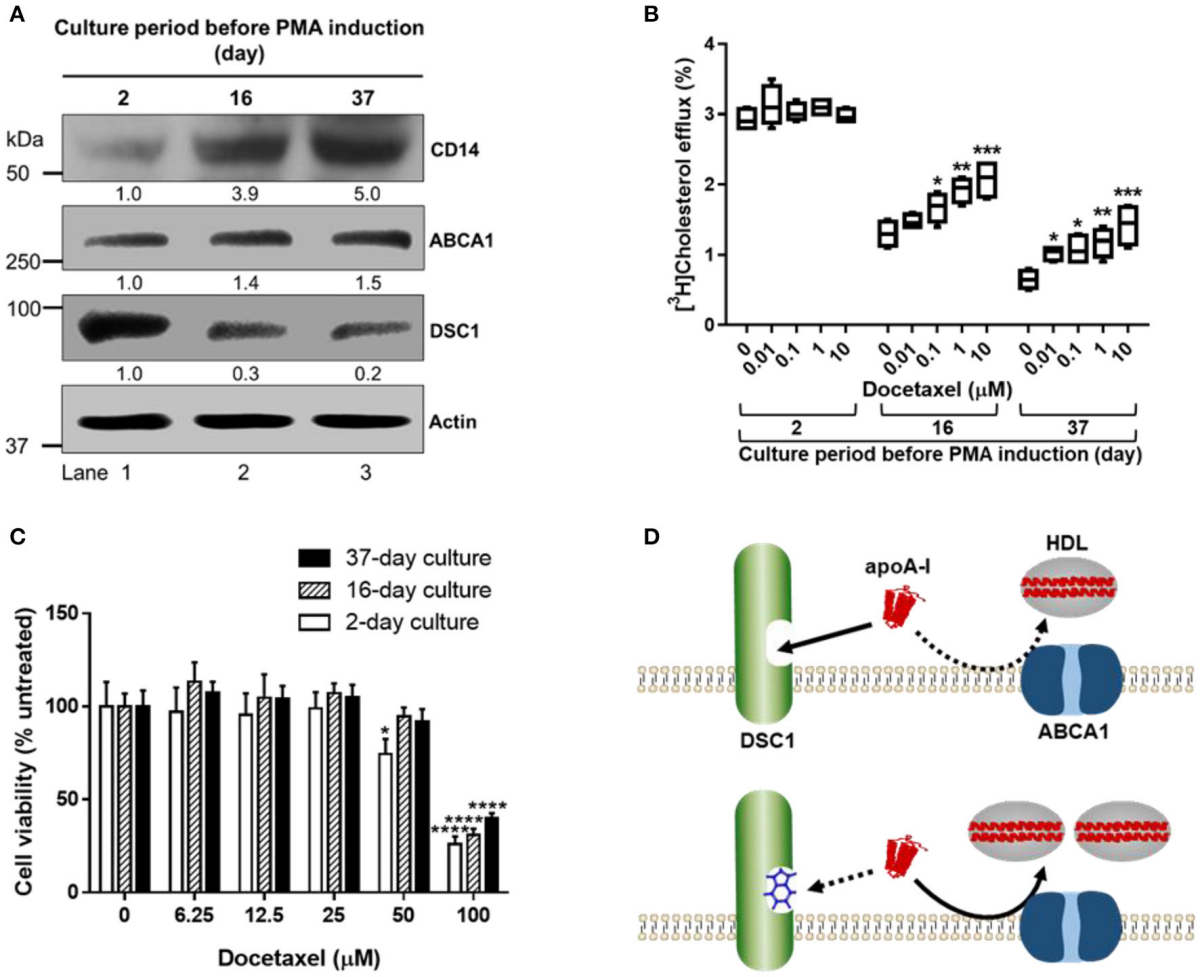
New strategies to promote ABCA1-mediated cholesterol efflux in differentiated macrophages. **(A)** THP-1 monocytes were cultured at the density of 2.0 × 10^6^/ml in RPMI 1640 medium supplemented with 10% fetal bovine serum and 100 IU penicillin and 100 mg/ml streptomycin. The culture medium was refreshed every 2–3 days (Mondays, Wednesdays, and Fridays) with counting/reseeding the cells at the density of 2.0 × 10^6^/ml every 3–4 days (Mondays and Fridays). The cells cultured for 2, 16, or 37 days were treated with 100 ng/ml of PMA for 3 days to differentiate monocytes into macrophages, washed with RPMI 1640 three times, rested for 2 days in the PMA-free culture medium, and lysed to determine protein expression levels by immunoblotting. Numeric values shown below the CD14, ABCA1, and DSC1 blots represent band densities normalized to actin and relative to the 2-day cultured cells (lane 1). **(B)** At the end of the 2 days resting period, THP-1 cells were incubated with 50 μg/ml of acetylated LDL and 1 μCi/ml of [^3^H]-cholesterol for 24 h, equilibrated for 24 h, and treated with 5 μg/ml of apoA-I for 6 h to measure ABCA1-mediated cholesterol efflux to apoA-I. The indicated concentrations of docetaxel were added during the equilibration and apoA-I treatment period (35). Results were expressed as percentage of total (cell plus medium) [^3^H]-sterol appearing in the medium. Data values were displayed by using a box and whisker plot (*n* = 4). One-way analysis of variance with Dunnett's *post-hoc* correction was performed to calculate multiplicity-adjusted *p*-values. ^*^p < 0.05; ^**^p < 0.01; ^***^p < 0.001 compared with the untreated control in each group. **(C)** At the end of the 2 days resting period, THP-1 cells were treated with indicated concentrations of docetaxel for 3 days prior to performing the sulforhodamine B cytotoxicity assay (35). Values are the mean ± standard deviation of triplicate determinations. Statistical analysis was performed as described above. ^*^p < 0.05; ^****^p < 0.0001 compared with the untreated control in each group. **(D)** DSC1 targeting strategies. The binding of apoA-I to DSC1 prevents ABCA1-mediated cholesterol efflux to apoA-I, thus small molecules blocking apoA-I-DSC1 interactions may hold therapeutic potential for reducing atherogenic cholesterol toxicity. In addition to small molecules such as docetaxel, peptide- and antibody-based targeting strategies may also be developed.

To investigate the effects of THP-1 culture density and period on the PMA-induced differentiation, we cultured THP-1 monocytes at a high density (2.0 × 10^6^/ml) in RPMI 1640 medium supplemented with 10% fetal bovine serum. With refreshing the medium every 2–3 days, THP-1 monocytes were cultured for 2, 16, or 37 days prior to treating the cells with PMA for their differentiation into macrophages. As PMA-induced differentiation protocols vary greatly, we went through a large body of literature and chose one of commonly used protocols: treatment of cells with 100 ng/ml of PMA for 3 days, followed by washing the cells with RPMI 1640 three times, followed by resting the cells for 2 days in PMA-free medium. At the end of the resting period, cells were harvested to determine protein expression levels. A co-receptor for bacterial lipopolysaccharide, CD14 is a key marker for macrophage function, and the upregulation of CD14 expression has been used as a readout of the differentiation of THP-1 monocytes into macrophages (46, 80). The CD14 expression level was proportional to the period of THP-1 monocyte culture, suggesting that the number of THP-1 monocytes sensitized by the high-density culture environment increases as a function of culture time (Figure 2A). The time-dependent increase in PMA-responsive THP-1 monocyte subpopulations generated CD14^low^ (lane 1), CD14^intermediate^ (lane 2) and CD14^high^ (lane 3) THP-1 cells (Figure 2A), which enabled us to study the effects of the three different degrees of differentiation on ABCA1-mediated cholesterol efflux. The CD14 levels were positively correlated with ABCA1 expression levels (Pearson's *r* = 0.9969) and negatively correlated with DSC1 expression levels (Pearson's *r* = −0.9881) (Figure 2A), suggesting that the differentiation process may modulate ABCA1-mediated cholesterol efflux. The marked decrease of DSC1 in CD14^intermediate^ (Figure 2A, lane 2) and CD14^high^ (Figure 2A, lane 3) THP-1 cells suggests that the functions of DSC1 may be greatly reduced during the differentiation and that DSC1 inhibition in differentiated macrophages may be achieved by using a low dose of DSC1-targeing drugs. We have recently reported that DSC1 binds apoA-I to prevent apoA-I from participating in the ABCA1-mediated cholesterol efflux pathway and that docetaxel promotes the ABCA1-mediated pathway by acting as a pharmacological inhibitor of apoA-I-DSC1 interactions (34, 35). To examine the relationship between effective docetaxel doses and DSC1 expression levels, we measured ABCA1-mediated cholesterol efflux from the three different THP-1 subtypes to apoA-I: the 2-day cultured CD14^low^DSC1^high^ THP-1 cells (Figure 2A, lane 1) showed no significant change in the cholesterol efflux in the presence of 0.01–10 μM docetaxel, whereas the 16-day cultured CD14^intermediate^DSC1^intermediatelow^ THP-1cells (Figure 2A, lane 2) and the 37-day cultured CD14^high^DSC1^low^ THP-1 cells (Figure 2A, lane 3) increased the cholesterol efflux in the presence of docetaxel in a dose-dependent manner (Figure 2B). In a separate experiment, we observed that 1 nM docetaxel was sufficient to significantly promote the cholesterol efflux in the 37-day cultured CD14^high^DSC1^low^ THP-1 cells. These results indicate that the DSC1 expression level is a factor determining effective docetaxel doses and that docetaxel may become a potent promoter of ABCA1-mediated cholesterol efflux in differentiated macrophages.

Despite being irresponsive to the docetaxel treatment, the CD14^low^DSC1^high^ THP-1 cells showed the highest capacity in ABCA1-mediated cholesterol efflux among the three THP-1 subtypes (Figure 2B). We observed that the CD14 expression levels were inversely associated with the ABCA1-mediated cholesterol efflux in THP-1 cells (Figure 2B). This is contrary to our expectations based on ABCA1 and DSC1 expression levels (Figure 2A), but the inverse relationship between the differentiation degree of THP-1 cells and ABCA1-mediated cholesterol efflux has been previously reported (50). The ABCA1-mediated cholesterol efflux to apoA-I was also reduced during the formation of THP-1 foam cells (50). These data suggest that differentiated macrophages may be impaired in ABCA1-mediated cholesterol efflux and that our current concept for macrophage cholesterol efflux might need to be reviewed. It has been thought that monocyte-derived macrophages and macrophage foam cells in the arterial wall activate the ABCA1-mediated cholesterol efflux pathway in order to alleviate cholesterol overload. If the ABCA1 pathway is compromised during the monocyte-to-macrophage differentiation, we may need to revise current strategies aimed at enhancing the pathway.

The cytotoxicity profile of docetaxel was also dependent on the THP-1 subtypes: 50 μM docetaxel was toxic in the 2-day cultured CD14^low^DSC1^high^ THP-1 cells but not in the 16-day cultured CD14^intermediate^DSC1^intermediatelow^ and the 37-day cultured CD14^high^DSC1^low^ THP-1 cells; 100 μM docetaxel was toxic in all the subtypes but the extent of toxicity was inversely correlated with the CD14 expression levels (Figure 2C). An anti-mitotic chemotherapy drug, docetaxel causes apoptotic cell death and PMA-differentiated THP-1 macrophages are resistant to apoptosis (39, 81). Thus, the inverse relationship between CD14 levels and susceptibility to docetaxel toxicity provides us with another evidence that the number of PMA-differentiated THP-1 macrophages increases in proportion to the period of the high-density THP-1 monocyte culture. As docetaxel is an anti-cancer agent, it is vital to determine the therapeutic window of docetaxel. ABCA1-mediated cholesterol efflux and cytotoxicity studies using the CD14^high^DSC1^low^ THP-1 cells suggest that the potency of docetaxel in promoting the cholesterol efflux may broaden its therapeutic window (Figures 2B,C). Monocyte-to-macrophage differentiation is an inevitable process during atherogenesis, therefore the most differentiated CD14^high^DSC1^low^ THP-1 cells may be an appropriate model to study macrophages in the atherosclerotic plaque. We observed that 50 μM docetaxel was not toxic in the CD14^high^DSC1^low^ THP-1 cells (Figure 2C) and that 1nM docetaxel was sufficient to significantly promote ABCA1-mediated cholesterol efflux in the cells, suggesting that anti-atherogenic doses of docetaxel may be several orders of magnitude lower than cytotoxic doses in macrophages. In cancer chemotherapy, patients receive weekly docetaxel doses of 35–40 mg/m^2^ or 3-weekly doses of 60–100 mg/m^2^ intravenously to achieve maximum plasma concentrations of 1.55–4.15 μg/ml (1.92–5.14 μM) (82, 83), which is at least 1,920 times higher than the cholesterol efflux dose 1nM. In addition, docetaxel showed a greater potency in promoting ABCA1-mediated cholesterol efflux in human skin fibroblasts (EC50 = 0.72 nM) and human aortic smooth muscle cells (EC50 = 0.35 nM) (35). These data suggest that docetaxel may become an effective anti-atherogenic agent with a wide margin of safety. Drug absorption, distribution, metabolism and excretion *in vivo* depend on multiple factors, thus pharmacokinetic studies to evaluate the action mechanism of docetaxel will be required for the delivery of anti-atherogenic doses of docetaxel into the subendothelial space of arteries.

The differentiation-associated decrease in ABCA1-mediated cholesterol efflux (Figure 2B) may force differentiated macrophages to increase their capacity to store cholesterol and thus to become foam cells. When the ABCA1-dependent formation of immature HDLs is reduced, other efflux pathways using immature and mature HDLs may also be affected (Figure 1). The promotion of ABCA1-mediated cholesterol efflux is therefore essential to improve cholesterol management in macrophages. We have shown that ABCA1-mediated cholesterol efflux to apoA-I is prevented by the binding of apoA-I to DCS1 and that the apoA-I binding site is in the extracellular domain of DSC1 (34, 35). Thus, small molecules that block the apoA-I binding site can promote ABCA1-mediated cholesterol efflux (Figure 2D). The identification of docetaxel as a potent blocker of apoA-I-DSC1 interactions is exciting in several aspects of drug development: (a) the localization of the DSC1 extracellular domain at the cell surface makes it an easy target to access, (b) reduced expression of DSC1 in differentiated macrophages improves pharmacological efficacy of docetaxel in blocking DSC1, and (c) the high potency of docetaxel allows to use low therapeutic doses and thus to reduce adverse side effects. Combined, these data advise us to develop docetaxel and docetaxel-derived compounds as new drugs for the promotion of ABCA1-mediated cholesterol efflux.

## Summary

Monocyte-derived macrophages are the primary component of atherosclerotic plaques and PMA-differentiated THP-1 cells have been most widely used to model human monocyte-derived macrophages. Although PMA has been effectively used to differentiate THP-1 monocytes into macrophages, the differentiation procedure is highly variable across studies, resulting in generating THP-1 cells in varying degrees of differentiation. To investigate the effects of the degree of THP-1 differentiation on ABCA1-mediated cholesterol efflux, THP-1 cells in low, intermediate and high degrees of differentiation were examined for their abilities to remove cholesterol to apoA-I. Even though ABCA1 expression levels increased and DSC1 expression levels decreased during the differentiation, ABCA1-mediated cholesterol efflux to apoA-I was inversely associated with the differentiation degree. These results suggest that the degree of differentiation may play a dominant role over ABCA1 and DSC1 in determining macrophage cholesterol efflux. DSC1, reduced in differentiated THP-1 macrophages, appears to be a viable drug target to promote ABCA1-mediated cholesterol efflux and was efficiently targeted by a small molecule, docetaxel. The potency, efficacy and a wide safety margin of docetaxel suggest that the ABCA1-mediated cholesterol efflux pathway may be therapeutically modulated. The blocking of the apoA-I binding site in DSC1 can be achieved by using small molecules, peptides and antibodies. These DSC1 targeting strategies may enable us to develop new therapies reducing cholesterol accumulation in the atherosclerotic plaque.

## Data Availability Statement

The raw data supporting the conclusions of this article will be made available by the authors, without undue reservation.

## Author Contributions

HC: conception and design, acquisition and analysis of data, and drafting the manuscript. IR: acquisition of data, reviewing, and editing the manuscript. SC: drafting the manuscript. JG: conception and design, analysis and interpretation of data, reviewing, and editing the manuscript. All authors read and approved the submitted version.

## Funding

This work was supported by the Canadian Institutes of Health Research grant PJT-165924 (to JG), which had no involvement in the study design, in the collection, analysis and interpretation of data, in the writing of the manuscript and in the decision to submit the article for publication.

## Conflict of Interest

HC and JG have filed Patent Cooperation Treaty (PCT) and United States (US) patent applications entitled “Desmocollin 1 Inhibitors for the Prevention or Treatment of Atherosclerosis” (PCT application no. PCT/CA2018/050669 and US patent application no. 16/619,789). The remaining authors declare that the research was conducted in the absence of any commercial or financial relationships that could be construed as a potential conflict of interest.

## Publisher's Note

All claims expressed in this article are solely those of the authors and do not necessarily represent those of their affiliated organizations, or those of the publisher, the editors and the reviewers. Any product that may be evaluated in this article, or claim that may be made by its manufacturer, is not guaranteed or endorsed by the publisher.

## Abbreviations

ASCVD: atherosclerotic cardiovascular diseases
LDL: low-density lipoprotein
HDL: high-density lipoprotein
VLDL: very-low-density lipoprotein
RCT: reverse cholesterol transport
ABCA1: ATP binding cassette A1 transporter
ABCG1: ATP binding cassette G1 transporter
SR-BI: scavenger receptor class B type I
AD: aqueous diffusion
LD: lipid droplet
PM: plasma membrane
apo: apolipoprotein
DSC1: desmocollin 1
PMA: phorbol 12-myristate 13-acetate
EC50: half-maximal effective concentration.
